# Left Stellate Ganglion Ablation Inhibits Ventricular Arrhythmias through Macrophage Regulation in Canines with Acute Ischemic Stroke

**DOI:** 10.7150/ijms.50976

**Published:** 2021-01-01

**Authors:** Youcheng Wang, Shanqing He, Xiaoxing Xiong, Jia Liu, Baojun Xie, Yajun Yao, Junkui Yin, Liuliu Zi, Xi Wang, Yanhong Tang, Qingyan Zhao

**Affiliations:** 1 Department of Cardiology, Renmin Hospital of Wuhan University, Cardiovascular Research Institute of Wuhan University, Hubei Key Laboratory of Cardiology, Wuhan City, Hubei Province, China; 2 Department of Neurosurgery, Renmin Hospital of Wuhan University, Wuhan City, Hubei Province, China; 3 Department of Radiology, Renmin Hospital of Wuhan University, Wuhan City, Hubei Province, China

**Keywords:** acute stroke, ventricular arrhythmia, sympathetic nerve, macrophage, canine

## Abstract

Aims: To investigate the potential mechanism of ventricular arrhythmias (VAs) after acute ischemic stroke and explore the effects of left stellate gangling (LSG) ablation on VAs induced by stroke in canines.

**Materials and Methods:** Twenty canines were randomly divided into the sham-operated group (n=6), AS group (n=7) and SGA group (n=7). Cerebral ischemic model was established in the AS group and the SGA group by right acute middle cerebral artery occlusion (MCAO). LSG ablation was performed in the SGA group as soon as MCAO. After 3 days, atrial electrophysiology and neural activity were measured in vivo. The levels of norepinephrine (NE) in plasma and ventricle were detected by ELISA. The levels of monocyte chemotactic protein-1 (MCP-1), tumor necrosis factor-α (TNF-α) and NF-κB p65 in ventricle were detected by western blotting. The pro-inflammatory polarization of macrophages in ventricle was detected by immunofluorescence.

**Results:** Higher ventricular tachycardia (VT) inducibility and lower ventricular fibrillation threshold (VFT) were observed in the AS group compared with those in the sham-operated group, associated with higher LSG activity and NE levels, increased number of M1 macrophages and secretion of inflammatory cytokines in ventricle (all P<0.001). Compared with the AS group, the SGA group had lower VT inducibility and higher VFT, combined with lower NE levels, and reduced number of M1 macrophages and secretion of inflammatory cytokines in ventricle (all P<0.001).

**Conclusion:** LSG ablation could reduce VAs vulnerability after acute stroke by preventing the macrophages polarization and activation induced by sympathetic hyperactivity.

## Introduction

The effects of cerebrovascular diseases on the modulation of autonomic nervous system and cardiovascular function have been studied for decades. Early observations showed that electrocardiographic (ECG) abnormalities and cardiac arrhythmias occurred in almost half of patients with an acute stroke 1. Although most patients died directly from the neurological injury during the first week after the stroke, about 2 to 6% of all stroke patients died from cardiac causes in the first 3 months and approximately 19% had fatal or serious non-fatal cardiac events 2. An increased risk of ventricular arrhythmias (VAs) is one of the most serious cardiac outcomes after stroke, leading to a high risk of sudden death 3. However, the exact mechanism of VAs and sudden death after acute stroke remains incompletely understood.

Previous studies have shown that acute stroke is often accompanied by autonomic dysfunction, especially sympathetic excessive activity, generally manifested as the increased blood pressure, a higher low frequency/high frequency ratio of heart rate variability, or the increased norepinephrine (NE) and cortisol levels in blood 4-6. It has been confirmed that the sympathetic excessive activity and the sympathetic nerve sprouting in myocardium are important factors leading to the occurrence of VAs in acute myocardial infarction 7. As is known to all, stellate ganglion (SG), which belongs to sympathetic ganglion in function, is an important pathway connecting sympathetic nerve and cardiac autonomic system. NE, the major neurotransmitter released from postganglionic sympathetic nerve terminals, can directly bind to β-adrenergic receptors on myocardial cell membrane, resulting in effective refractory period (ERP) and action potentials shortening, and cardiac automaticity increasing. Meanwhile, NE is also known to be a potential pro-inflammatory mediator and can induce the release of multiple inflammatory factors 8, 9. Previous studies have suggested that methods inhibiting the sympathetic activity, such as stellate ganglion ablation (SGA) and renal sympathetic denervation, have an positive effect on the myocardial electrophysiological changes and can prevent the occurrence of VAs during acute myocardial infarction 10, 11. In this study, we investigated the potential mechanism of VAs after acute ischemic stroke and explored the effects of SGA on VAs induced by stroke in canines.

## Materials and methods

This study was approved by the animal studies subcommittee of our institutional review board and was in compliance with the guidelines of the National Institutes of Health for the care and use of laboratory animals.

### Animal model preparation

An intramuscular injection of 25 mg/kg ketamine sulfate was administered before pentobarbital sodium (ASPEN Biotechnology Co., Ltd, China) premedication. All of the canines were premedicated with sodium pentobarbital (30 mg/kg, IV), intubated, and ventilated with room air supplemented with oxygen from a respirator (MAO01746, Harvard Apparatus Holliston, USA). Continuous ECG monitoring was performed. Twenty beagle canines (weighing 9 to 10 kg) were randomly assigned to 3 groups. The sham-operated group consisted of 6 canines that underwent craniotomy without right middle cerebral artery occlusion (MCAO). The AS group consisted of 7 canines in which a cerebral ischemic model was established through the occlusion of the right middle cerebral artery. The SGA group consisted of 7 canines that underwent the left stellate ganglion (LSG) ablation as soon as MCAO was completed.

All the operations followed the principle of sterility, and prophylactic antibiotics were given in the perioperative period.

### MCAO

**Figure 1:**
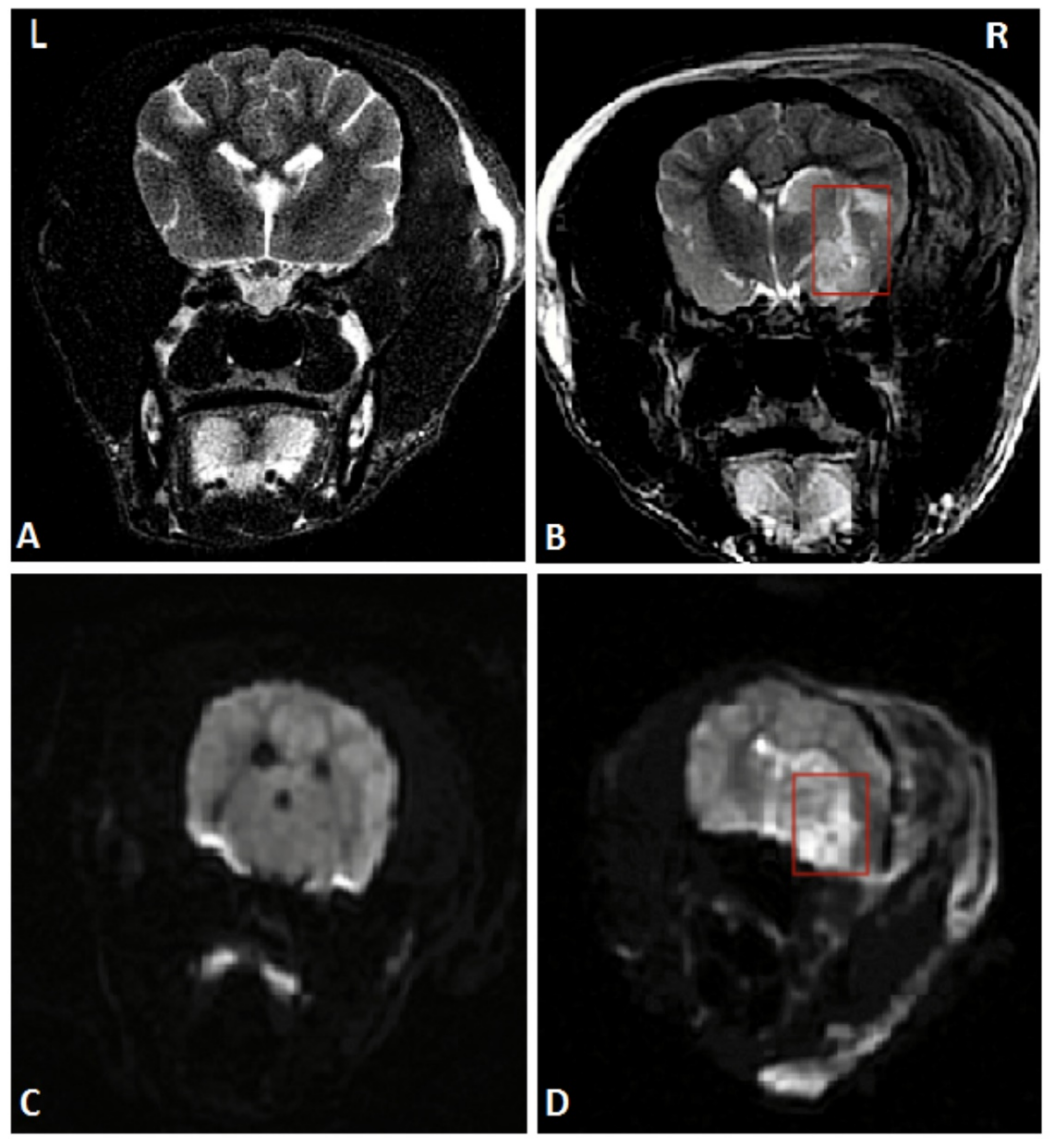
A head MRI that was performed 24 hours after MCAO suggested the right temporal lobe infarction, marked by red rectangles. (A) and (C) Coronal MRI scan of the sham-operated group in T2WI and DWI respectively. (B) and (D) Coronal MRI scan of the AS group in T2WI and DWI respectively. **Abbreviations:** AS: acute stroke; SGA: stellate ganglion ablation; MCAO: middle cerebral artery occlusion; MRI: magnetic resonance imaging; T2WI: T2 weighted imaging; DWI: diffusion weighted imaging.

To establish the model of acute ischemic stroke, MCAO on canines was performed. After successful anesthesia, right craniotomy was performed to expose the trunk of the right middle cerebral artery under a microscope. The trunk was stanched by bipolar electrocoagulation and then cut off. Detailed operational procedures were shown in the previous study 12. Magnetic resonance imaging (MRI) of the head was performed 24 hours after the operation to ensure the success of the stroke model. The MRI images are shown in **Figure 1.**

### LSG ablation

The LSG was completely exposed by left thoracotomy through the second intercostal space. A radiofrequency current (30-35 W, 150 s) was delivered to the site by an electrode catheter (Biosense Webster, Inc, Diamond Bar, CA) showing blood pressure (BP) elevation during stimulation. Complete ablation was verified by the abolishment of BP elevation during the delivery of electrical stimulation to the ablated site.

### Recording of LSG activity

LSG activity was measured in the sham-operated group and the AS group 3 days after the operations. To avoid the disturbance of electrical stimulation, the recordings of autonomic neural activity were performed before the electrophysiological measurements. Pairs of bipolar hook electrodes (Xi'an Friendship Medical Electronics Co., Ltd., China) were attached to the LSG. All measured signals were amplified and filtered between 0.3 Hz and 1 kHz with a PowerLab system (AD Instruments, Dunedin, New Zealand). Neural signals that were three times higher than noise signal were marked. Neural activity was represented by the frequency (per minute) and amplitude of neural discharges.

### Electrophysiological measurements

The stimuli were delivered with a computerized electrophysiology system (Lead 7000, Jinjiang Inc., China). ERPs in the right ventricular epicardium at the apex (RVA), free wall (RVFW), base (RVB) and left ventricular epicardium at the apex (LVA), free wall (LVFW), base (LVB) were determined during ventricular pacing at 300 ms cycle length using stimuli at twice threshold. A single premature stimulus (S2) was introduced after 8 basic stimuli (S1), starting with a coupling interval of 250 ms and reducing by 10 ms decrement. As the S1-S2 intervals approached the ERP, decrement was reduced to 2 ms. ERP was the longest S1-S2 interval at which S2 failed to capture. ERP dispersion (dERP) was the maximum difference among all sites tested.

ECG was continuously recorded for 1 hour to calculate the number of spontaneous VAs. VAs were classified as ventricular premature beat (VPB), ventricular tachycardia (VT; ≥3 consecutive ventricular premature beats), and ventricular fibrillation (VF).

Inducibility of VAs was assessed with programmed ventricular stimulation from right free wall. Single (S2) and double (S3) extrastimuli were delivered after 8 beats of ventricular drive (S1) at 300 ms (twice threshold, 2 ms duration). Stimulation was terminated when VT or VF was induced.

The ventricular fibrillation threshold (VFT) was measured at the end of the study. VF was evoked by programmed electrical stimulation at the right ventricular epicardium (a fixed train of 30 stimuli, 30 ms interval). The VFT was determined by the progressively increasing pacing voltage from 1 V, with a 30 s rest period before the next pacing train if no VF was induced. VF was defined as chaotic, fractionated electrical activity persisting for >5 s and then was electrically defibrillated to the sinus rhythm.

### Enzyme-linked immunosorbent assay (ELISA)

Blood samples from jugular vein separately obtained at baseline and 3 days after operations and tissue specimens that were obtained from the left ventricle (LV) were temporarily stored at -80 °C until the assay. Then, samples from all groups were assigned to measure NE levels examined by General NE ELISA Kit (ELK Biotechnology, China), based on the protocol provided by the manufacturer.

### Immunofluorescence

At the end of measurements, the ventricular tissues were quickly removed and fixed in 4% paraformaldehyde, embedded in paraffin and sectioned at 5 microns. To identify the pro-inflammatory M1 macrophages, the ventricular samples were incubated with primary antibodies against CD68 (1:100, Abcam, Inc., UK) and inducible nitric oxide synthase (iNOS; 1:50, Abcam, Inc., UK), and cells were stained with DAPI for nuclei. The results were analyzed by Image-Pro Plus 6.0 software. More than 3 sections of ventricle from different samples were randomly selected to quantify the number of macrophages.

### Real-time PCR

The expression of iNOS in ventricle was measured by real-time PCR. Total RNA was isolated from ventricular samples with Tripure Extraction Reagent (ELK Biotechnology, China) according to the manufacturer's protocol. cDNA was synthesized using EntiLink™ 1st Strand cDNA Synthesis Kit (ELK Biotechnology, China). Real-time fluorescent quantitative PCR was performed on the StepOne real-time PCR instrument (Life technologies, Gaithersburg, MD), and each sample was made into three duplications using EnTurbo™ SYBR Green PCR SuperMix kit (ELK Biotechnology, China). Primers used for RT-PCR were: Dog-iNOS: 5'-ACCAATACAGGCTCGTGCAG-3'(forward),5'-GGGCTGTCTACTACTCGCTCC-3'(reverse);Dog-GAPDH:5'-GAAGGTCGGAGTGAACGGATT-3'(forward),5'-CATTTGATGTTGGCGGGATC-3'(reverse).

### Western blotting

The membranes were incubated with a primary antibody against monocyte chemotactic protein-1 (MCP-1; Abcam, Inc., UK), tumour necrosis factor-alpha (TNF-α; Abcam, Inc., UK), NF-κB p65 (Abcam, Inc., UK). The membranes were blocked with 5% nonfat dry milk in Tris-buffered saline with Tween 20 (TBST) for 1 hour and incubated with the primary antibody overnight at 4 °C. They were then washed in TBST three times, incubated with the secondary antibody for 1 hour at 37 °C, and imaged using Immun-Star horseradish peroxidase substrate. The relative expression levels of the proteins were determined using image analyzer software (AlphaEase FC, San Leandro, CA, USA).

### Statistical analysis

The data were expressed as the mean ± standard deviation. Two-sample independent Student's t-tests were used to compare the means of two groups. ANOVA followed by Newman-Keuls tests was used to compare the mean values of continuous variables among multiple groups, and any significant differences were further analyzed using the Tukey-Kramer test. All the statistical tests were two-sided, and a probability value <0.05 was required for statistical significance.

## Results

### Measurements of ventricular ERPs and dERP

**Figure 2:**
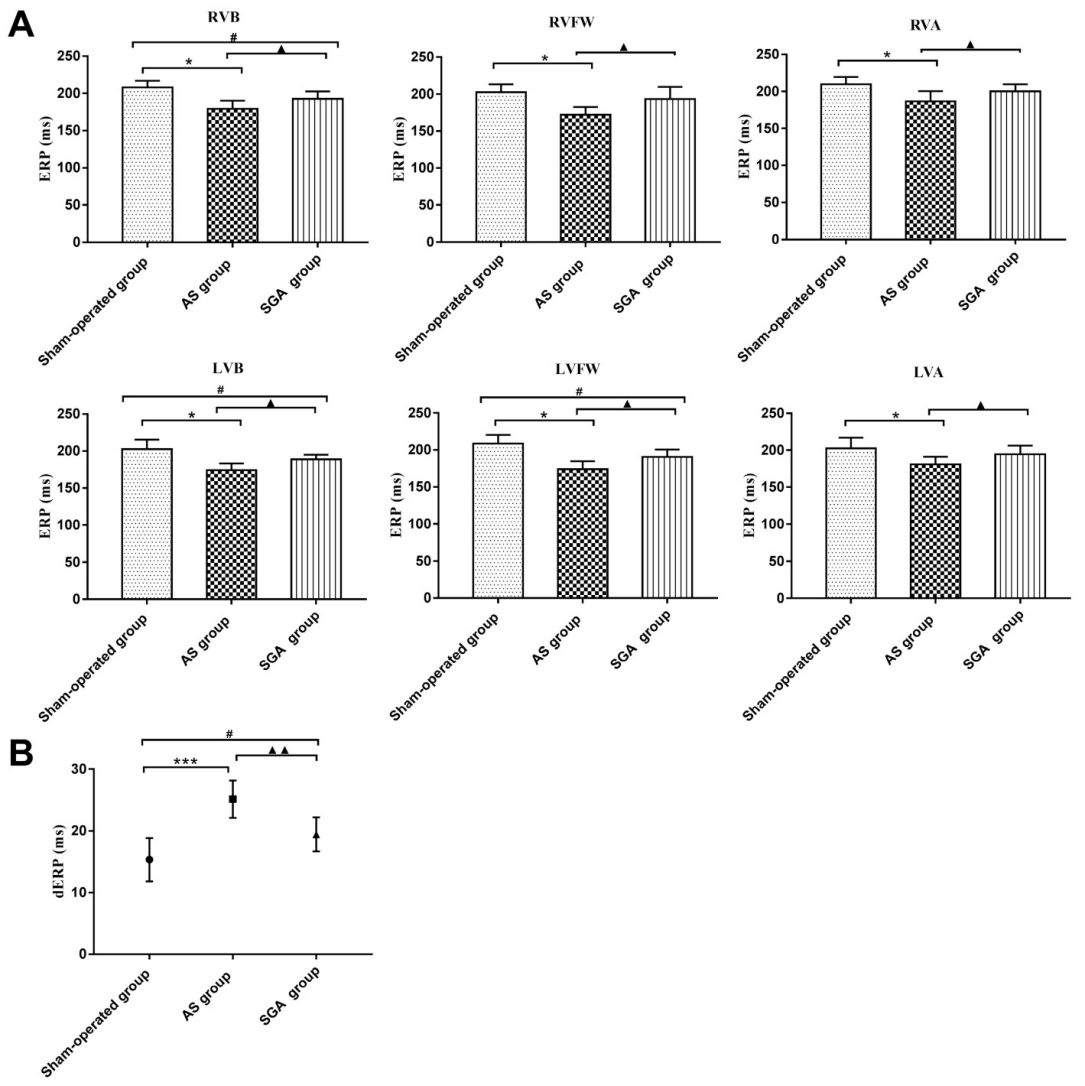
Differences in (A) the ventricular ERPs and (B) the dERP in all the groups. (A) The ERPs at all recording sites in the AS group were significantly reduced than those in the sham-operated group and the SGA group. The ERPs at RVB, LVB and LVFW were lower in the SGA group than those in the sham-operated group. However, there were no significant difference in ERPs at RVFW, RVA and LVA between the sham-operated group and the SGA group. *P<0.05; ^#^P<0.05; ^▲^P<0.05. (B) The dERP in the AS group was significantly increased than that in the sham-operated group and the SGA group. The dERP was higher in the SGA group than that in the sham-operated group. ***P<0.001; ^#^P<0.05; ^▲▲^P<0.01. **Abbreviations:** AS: acute stroke; SGA: stellate ganglion ablation; ERPs: effective refractory periods; dERP: dispersion of effective refractory period; RVA: right ventricular epicardium at the apex; RVFW: right ventricular epicardium at the free wall; RVB: right ventricular epicardium at the base; LVA: left ventricular epicardium at the apex; LVFW: left ventricular epicardium at the free wall; LVB: left ventricular epicardium at the base.

As shown in **Figure 2A**, the ERPs at all recording sites in the AS group were significantly reduced than those in the sham-operated group and the SGA group (all P<0.05). For example, the ERP at RVB was 179 ± 11 ms in the AS group, while the ERP at RVB was 208 ± 9 ms in the sham-operated group and 193 ± 10 ms in the SGA group. Compared with those in the sham-operated group, the ERPs at RVB, LVB and LVFW were lower in the SGA group (P<0.05). However, there were no significant differences in ERPs at RVFW, RVA and LVA between the sham-operated group and the SGA group.

As shown in **Figure 2B**, the dERP in the AS group was significantly increased than that in the sham-operated group (25 ± 3 versus 15 ± 4 ms, P<0.001) and the SGA group (25 ± 3 versus 19 ± 3 ms, P<0.01). The dERP was higher in the SGA group (P<0.05) than that in the sham-operated group.

### Recording of Spontaneous VAs

**Figure 3:**
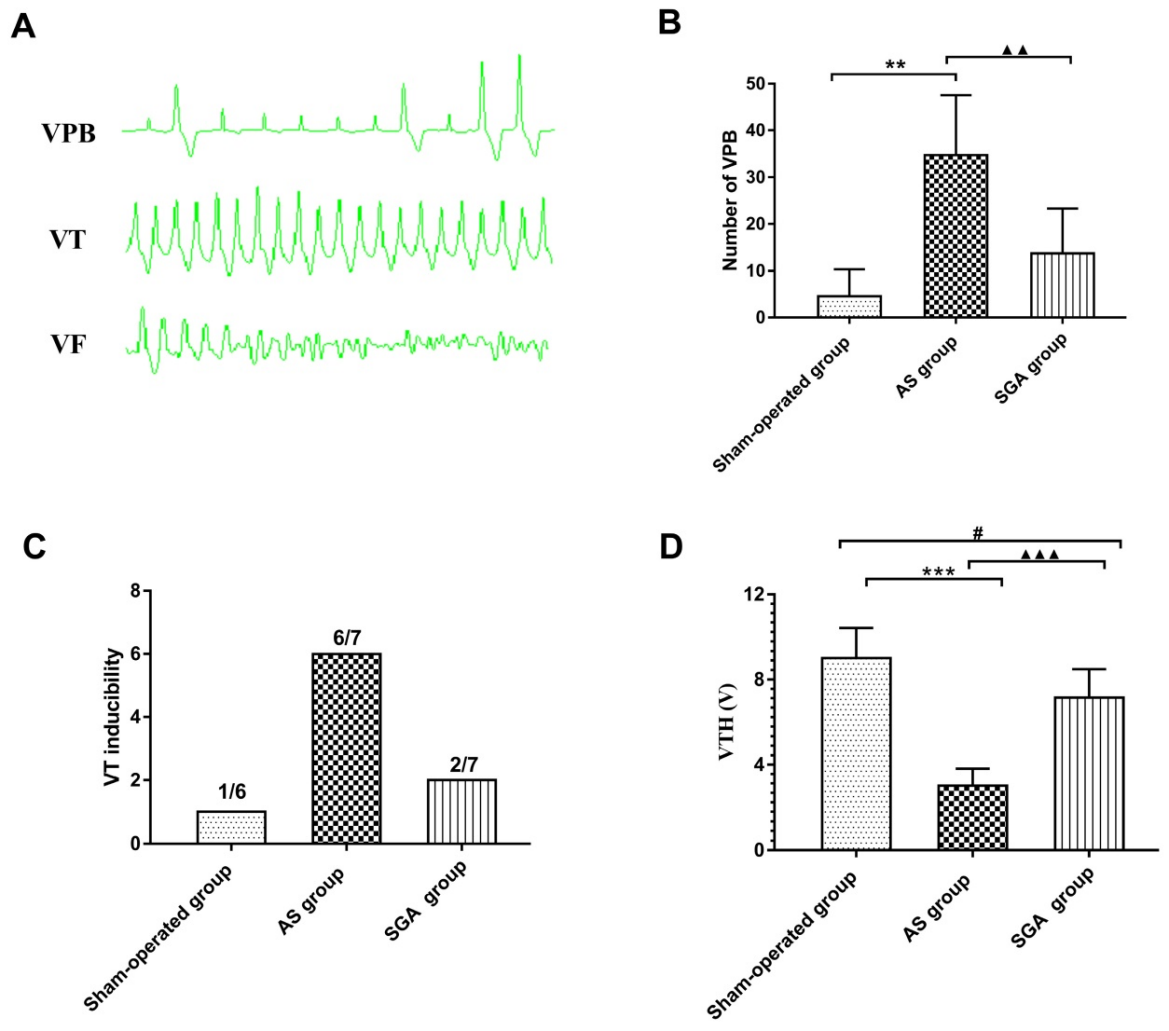
The number of (B) spontaneous VPB, (C) VT inducibility and (D) VFH in all the groups. (A) Examples of VPB, VT and VF occurrence. (B) The number of spontaneous VPB during the monitoring was increased than that in the sham-operated group and the SGA group. The SGA group had a higher number of VPB than the sham-operated group, but there was no statistical significant. **P<0.01; ^▲▲^P<0.01. (C) VT was induced in 1 canine in the sham-operated group (1/6), 6 canines in the AS group (6/7) and 2 canines in the SGA group (2/7). No VF was induced in all the groups. (D) The AS group had an significantly reduced VFH than the sham-operated group and the SGA group. Compared with that in the sham-operated group, the VFH in the SGA group was lower. ***P<0.001;^ #^P<0.05; ^▲▲▲^P<0.001. **Abbreviations:** AS: acute stroke; SGA: stellate ganglion ablation; VPB: ventricular premature beat; VT: ventricular tachycardia; VF: ventricular fibrillation; VFH: ventricular fibrillation threshold.

During the ECG monitoring, no spontaneous VF was observed in all groups. Only one canine in the AS group had one short episode of VT. The number of spontaneous VPB in the AS group was significantly increased than that in the sham-operated group (34 ± 13 versus 4.5 ± 6, P<0.01) and the SGA group (34 ± 13 versus 14 ± 10, P<0.01). Compared with the sham-operated group, an increasing trend of number of VPB in the SGA group was observed, but there was no statistical significance (**Figure 3B**).

### Measurements of VAs inducibility and VFT

As shown in **Figure 3C**, the programmed ventricular stimulation induced one short episode of VT in only one canine in the sham-operated group. In the AS group, the short episode of VT was induced in four canines, and sustained VT was induced in two canines. In the SGA group, the short episode of VT was induced in two canines. There was no VF induced in all the groups. As shown in **Figure 3D**, VFT in the AS group was significantly reduced than that in the AS group ( 3.0 ± 0.8 versus 9.0 ± 1.4 V, P<0.001) and the SGA group (3.0 ± 0.8 versus 7.1 ± 1.3 V, P<0.001). Compared with that in the sham-operated group, the VFT was lower in the SGA group (P<0.05).

### Recording of LSG activity

**Figure 4:**
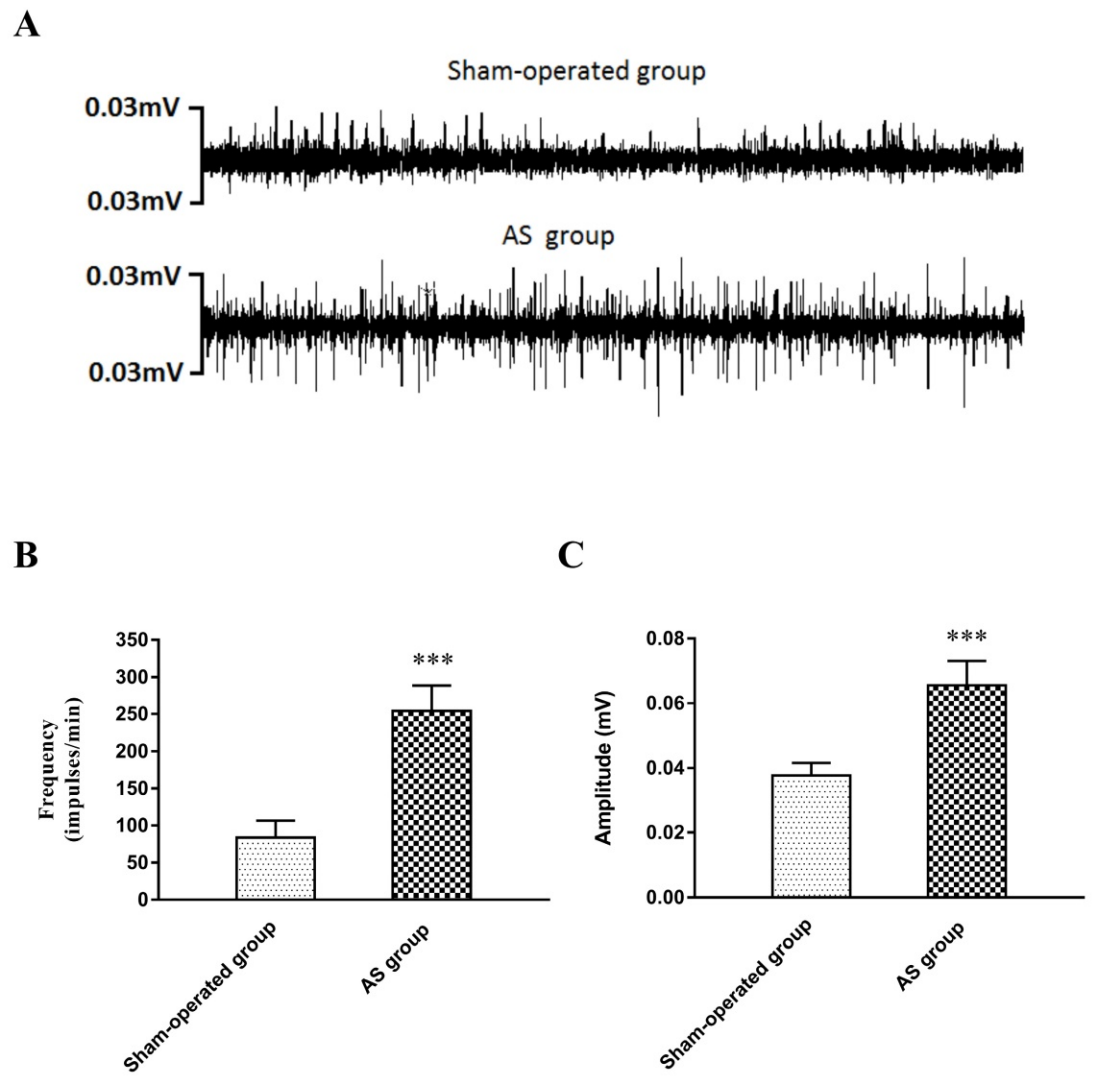
(A) Examples of LSG activity 3 days after operation. (B) The frequency and (C) the amplitude in the AS group were greatly elevated compared with those in the sham-operated group. ***P<0.001. **Abbreviations:** LSG: left stellate ganglion; AS: acute stroke.

The neural activity of the LSG was recorded in the sham-operated group and the AS group 3 days after the operation. The results are shown in **Figure 4**. Compared with those in the sham-operated group, the frequency and amplitude of the LSG were significantly elevated in the AS group (frequency: 254 ± 34 versus 83 ± 23 impulses/min,* P*<0.001; amplitude: 0.0656 ± 0.008 versus 0.0377 ± 0.004 mV, *P*<0.001).

### Measurements of NE levels in plasma and ventricle

As shown in **Table 1**, the levels of NE in plasma and LV were significantly elevated in the AS group 3 days after MCAO. LSG ablation could suppress the elevated NE levels induced by acute stroke.

### Pro-inflammatory macrophages polarization in ventricle

**Figure 5:**
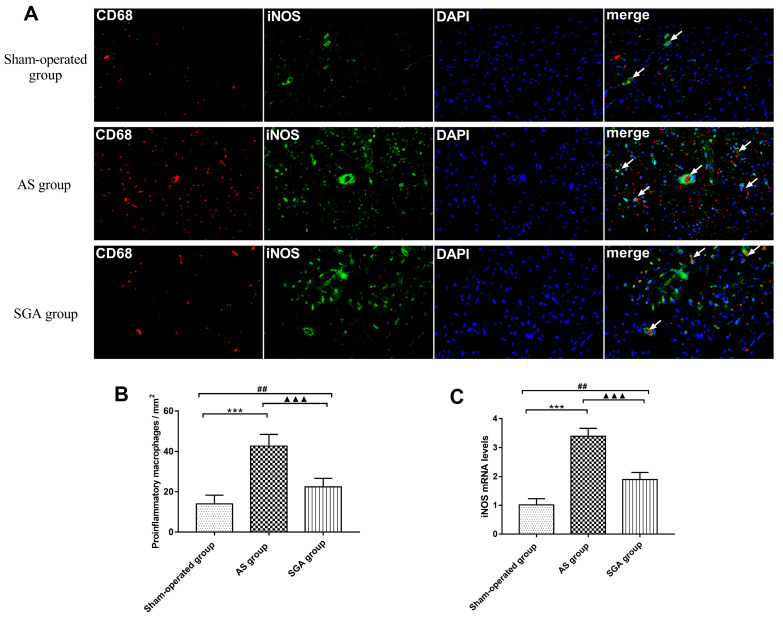
(A) The immunofluorescence of pro-inflammatory M1 macrophages in ventricle. (B) the number of pro-inflammatory M1 macrophages in ventricle was significantly increased in the AS group than that in the sham-operated group and the SGA group. The SGA group had a higher number of M1 macrophages in ventricle compared with the sham-operated group. (C) The relative levels of iNOS mRNA in ventricle were significantly increased in the AS group than those in the sham-operated group and the SGA group. The SGA group had higher levels of iNOS mRNA in ventricle compared with the sham-operated group. ***P<0.001; ^##^P<0.01; ^▲▲▲^P<0.001. **Abbreviations:** AS: acute stroke; SGA: stellate ganglion ablation; iNOS: inducible nitric oxide synthase.

As shown in **Figure 5A** and** 5B**, the number of pro-inflammatory M1 cells in ventricle was significantly increased in the AS group, compared with that in the sham-operated group (42 ± 6 versus 14 ± 4 per mm^2^, P<0.001) and the SGA group (42 ± 6 versus 22 ± 4 per mm^2^, P<0.001). The number of M1 cells in ventricle was higher in the SGA group than that in the sham-operated group (P<0.01). Real-time PCR of iNOS was also used for further clarification (**Figure 5C**). In the AS group, the relative levels of iNOS mRNA in ventricle were markedly elevated than those in the sham-operated group (3.39 ± 0.27 versus 1.01 ± 0.21, P<0.001) and the SGA group (3.39 ± 0.27 versus 1.89 ± 0.25, P<0.001). The SGA group had higher levels of iNOS mRNA in ventricle compared with the sham-operated group (P<0.001).

### Levels of inflammatory cytokines in ventricle

**Figure 6:**
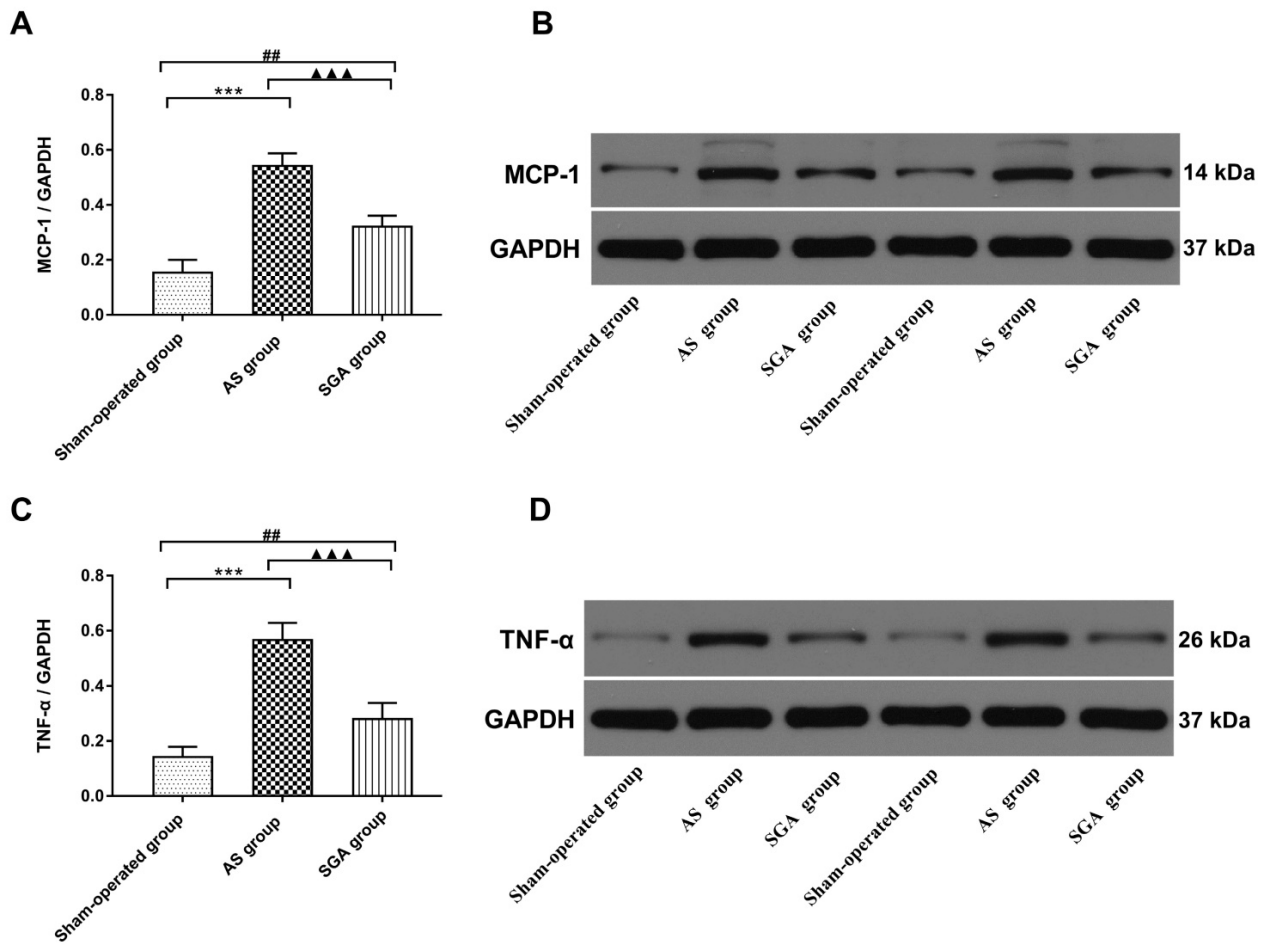
(A) The levels of MCP-1 and (D) TNF-α in ventricle were significantly increased in the AS group, compared with those in the sham-operated group and the SGA group. The SGA group had higher levels of MCP-1 and TNF-α in ventricle than the sham-operated group. ***P<0.001; ^##^P<0.01; ^▲▲▲^P<0.001. (B) and (D) The Western blotting results of MCP-1 and TNF-α in ventricle. **Abbreviations:** AS: acute stroke; SGA: stellate ganglion ablation; MCP-1: monocyte chemotactic protein-1; TNF-α: tumour necrosis factor-alpha.

**Figure 7:**
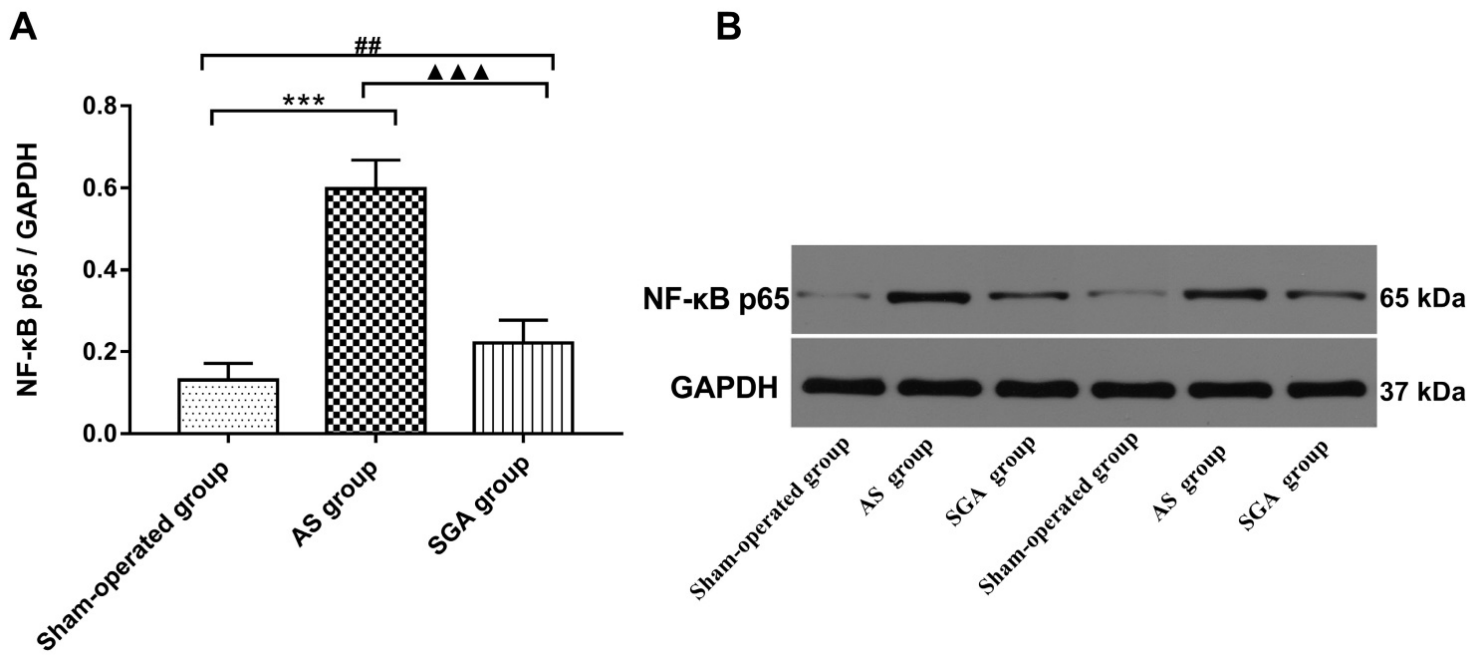
(A) The expression of NF-κB P65 protein in ventricle was significantly elevated in the AS group, compared with that in the sham-operated group and the SGA group. The SGA group had a higher expression of NF-κB P65 protein in ventricle than the sham-operated group. ***P<0.001; ^##^P<0.01; ^▲▲▲^P<0.001. (B) The Western blotting results of NF-κB P65 protein in ventricle. **Abbreviations:** AS: acute stroke; SGA: stellate ganglion ablation.

All immunoblot band intensity measurements were normalized to the intensity of the GAPDH band in the loaded sample. As shown in **Figure 6**, the levels of MCP-1 and TNF-α in ventricle were markedly increased in the AS group, compared with those in the sham-operated group (TNF-α: 0.57 ± 0.06 versus 0.14 ± 0.04; MCP-1: 0.54 ± 0.05 versus 0.15 ± 0.05; P<0.001) and the SGA group (TNF-α: 0.57 ± 0.06 versus 0.28 ± 0.06; MCP-1: 0.54 ± 0.05 versus 0.32 ± 0.05; P<0.001). The levels of MCP-1 and TNF-α in ventricle were higher in the SGA group than those in the sham-operated group (P<0.01). As shown in **Figure 7**, the expression of NF-κB p65 protein in ventricle was markedly higher in the AS group, compared with that in the sham-operated group (0.59 ± 0.07 versus 0.13 ± 0.04, P<0.001) and the SGA group (0.59 ± 0.07 versus 0.22 ± 0.06, P<0.001). The expression of NF-κB p65 in ventricle was higher in the SGA group than that in the sham-operated group (P<0.01).

## Discussion

This study investigated the influence of SGA on VAs in a canine model of MCAO and its potential mechanism. We provided evidence of the following: (1) An acute ischemic stroke can lead to the increased risk of VAs, which may be associated with the pro-inflammatory polarization of macrophages induced by sympathetic excessive activity; and (2) LSG ablation can inhibit the occurrence of VAs after an acute stroke by downregulating the catecholamine levels in plasma and preventing macrophages activating in myocardium.

Abnormal ECGs and cardiac arrhythmias are commonly identified in patients with acute stroke, varying from abnormal T waves, to QT prolongation, to fatal arrhythmias, such as VF that causes sudden cardiac death 13. The previous clinical studies have suggested that, acute strokes, especially involving the insular cortex, increase the incidence of VAs through a cascade of events that alter autonomic balance and increase catecholamine levels. Besides VAs, the typical myocardial damage induced by acute stroke has also been commonly observed 14, 15. The interval between the stroke onset and the consequence of ventricular arrhythmogenesis has not been clarified because the long-term studies with large sample sizes are lacking. According to the available studies, new VAs are mostly detected within 3 days after the stroke onset 16, 17. In the present study, although we failed to perform the postoperative continuous ECG monitoring, we found that the number of spontaneous VPB was markedly increased in the AS group during the 1 hour ECG monitoring. Besides, the AS group showed significantly reduced ERPs and TFV, increased dERP and VT inducibility at programmed electrical stimuli 3 days after the MCAO.

Clinical and experimental researches have shown that autonomic dysfunction is commonly observed after an acute stroke, mainly manifested as sympathetic overactivation 5, 6. The sympathetic excessive activity and sympathetic nerve sprouting in myocardium have been demonstrated to play important roles in VAs vulnerability. By contrast, the decreased sympathetic nerve activity such as renal denervation or LSG inhibition may have assisted in suppressing VAs. Anatomically, the majority of cardiac sympathetic axons and nerve terminals originate in the cell bodies of the SG. It is well known that the LSG highly involves in the regulation of ventricular electrophysiology. Yu et al. 18 found that LSG inhibition could stabilize cardiac electrophysiology and suppress susceptibility to ischemia induced VAs in canines. Vaseghi et al. 19 demonstrated that LSG denervation could effectively decrease sustained VT and implantable cardioverter-defibrillator shock recurrence in patients with refractory VT. In the present study, we found that neural activity of the LSG significantly increased, combined with elevated serum levels of NE in canines with acute stroke. LSG ablation could significantly attenuate the shortened ERPs and the increased VAs vulnerability caused by acute ischemic stroke.

The previous studies have demonstrated that inflammatory responses are involved in the pathogenesis of VAs induced by myocardial infarction via degradation connexin 43 (CX43) 20, and the enhanced inflammatory responses are associated with LSG remodeling through sympathetic nerve activation 21. Furthermore, macrophages activation as well as pro-inflammatory M1 phenotype polarization plays a crucial role in mediating infarction-related inflammatory responses and electrical conduction in the heart 22, 23. A recent histopathologic study documented that the infiltration of macrophages was much higher in the myocardium of patients who died after subarachnoid hemorrhage 24. However, the association between the cardiac macrophages activation and the acute ischemic stroke has not been well explored. In the present study, we found that the infiltration of M1 macrophages was increased, combined with the elevated levels of MCP-1 and TNF-α in ventricle in canines with acute ischemic stroke, which could be attenuated by LSG ablation.

It has been demonstrated that NE is a potential pro-inflammatory mediator and can induce the secretion of inflammatory cytokines via macrophages activation 8. Li et al. 9 have demonstrated that NE is capable of inducing IL-6 production in macrophages via the β-adrenoreceptor-NAD(P)H oxidase system-NF-κB signaling pathway. The NF-κB signaling pathway promotes pro-inflammatory M1 polarization and plays an essential role in macrophage-initiated inflammatory responses 25. In the present study, we found that the level of NF-κB p65 in ventricle elevated after acute ischemic stroke and that LSG ablation could reduce NF-κB p65 expression. These results suggested that sympathetic excessive activity after acute stroke induced M1 macrophages polarization and inflammatory chemokines secretion possibly via the NF-κB signaling pathway.

## Conclusions

In our study, we demonstrated that sympathetic hyperactivity caused by acute stroke could lead to the increased VAs vulnerability through the macrophages polarization and the secretion of inflammatory cytokines. LSG ablation could suppress the activity of macrophages and thereby inhibit VAs occurrence after acute stroke.

### Study limitations

This study has several limitations. Firstly, in this study, we failed to conduct postoperative continuous ECG monitoring to record the spontaneous VAs after acute stroke. Secondly, we did not measure the action potential duration (APD) and monophasic action potential (MAP) in endocardium and epicardium. Thirdly, we failed to investigate the effects of bilateral SGA on stroke induced VAs for comparison. Finally, previous studies have suggested the asymmetry of the bilateral insular cortex in the regulation of the autonomic nervous system. We did not explore the effects of different brain lesion locations on VAs. Whether damages of different areas have different influences on VAs remains unknown.

## Figures and Tables

**Table 1:** Difference of NE levels in plasma and LV

	Sham-operated group		AS group		SGA group	
**NE** (pg/ml)	base	3 days later	base	3 days later	base	3 days later
**plasma**	431.7±53.8	451.1±43.5	436.2±46.4	692.8±44.4***	419.4±45.2	542.1±50.1^###▲▲^
**LV**		139.9±21.7		351.8±30.3***		217.5±25.5^###▲▲^

***P<0.001 versus the sham-operated group at the same time; ^###^P<0.001 versus the AS group at the same time; ^▲▲^P<0.01 versus the sham-operated group at the same time. **Abbreviations:** AS: acute stroke; SGA: stellate ganglion ablation; NE: norepinephrine; LV: left ventricle.
